# The Angiotensin-Melatonin Axis

**DOI:** 10.1155/2013/521783

**Published:** 2013-01-08

**Authors:** Luciana A. Campos, Jose Cipolla-Neto, Fernanda G. Amaral, Lisete C. Michelini, Michael Bader, Ovidiu C. Baltatu

**Affiliations:** ^1^Center of Innovation, Technology and Education—(CITE), Camilo Castelo Branco University (UNICASTELO), São José dos Campos Technology Park, Presidente Dutra Road Km 138, 12247-004 São José dos Campos, SP, Brazil; ^2^Department of Physiology, Institute of Biomedical Sciences, University of São Paulo, 05508-000 São Paulo, SP, Brazil; ^3^Cardiovascular Research, Max Delbruck Center for Molecular Medicine, 13125 Berlin, Germany

## Abstract

Accumulating evidence indicates that various biological and neuroendocrine circadian rhythms may be disrupted in cardiovascular and metabolic disorders. These circadian alterations may contribute to the progression of disease. Our studies direct to an important role of angiotensin II and melatonin in the modulation of circadian rhythms. The brain renin-angiotensin system (RAS) may modulate melatonin synthesis, a hormone with well-established roles in regulating circadian rhythms. Angiotensin production in the central nervous system may not only influence hypertension but also appears to affect the circadian rhythm of blood pressure. Drugs acting on RAS have been proven effective in the treatment of cardiovascular and metabolic disorders including hypertension and diabetes mellitus (DM). On the other hand, since melatonin is capable of ameliorating metabolic abnormalities in DM and insulin resistance, the beneficial effects of RAS blockade could be improved through combined RAS blocker and melatonin therapy. Contemporary research is evidencing the existence of specific clock genes forming central and peripheral clocks governing circadian rhythms. Further research on the interaction between these two neurohormones and the clock genes governing circadian clocks may progress our understanding on the pathophysiology of disease with possible impact on chronotherapeutic strategies.

## 1. Introduction

The renin-angiotensin system (RAS) is considered as a major endocrine regulator of cardiovascular homeostasis. The RAS acts in endocrine, paracrine, and autocrine manner in several organs and systems exercising various organ-specific actions with effects on the cardiovascular system [1]. Several lines of evidence from integrative physiology and functional genomics to molecular and genetic levels indicate that the RAS, circulating (endocrine) or tissue (paracrine and autocrine), is one of the major drivers of hypertension and cardiovascular diseases [1–3]. This knowledge led to the successful development of drugs to block the RAS system (angiotensin converting enzyme inhibitors, angiotensin receptor blockers, and renin inhibitors) that proved efficacious in the treatment of hypertension and other cardiovascular diseases [4].

Melatonin is produced by the pineal gland predominantly during night and it is considered as a major hormone regulating the circadian rhythmicity of several biological systems [5]. Research on melatonin functions revealed that this is not only a regulator of the biological circadian clock [5] but also it has a variety of biological functions [6]. Melatonin appears to be involved in various diseases, such as sleep disorders, dementia, mood disorders, cancer, and diabetes [7].

Both angiotensin and melatonin are synthesized in the brain. Angiotensin produced locally in central nervous system in nuclei involved in cardiovascular and fluid-electrolyte homeostasis interacts with other systems, such as sympathetic, vasopressinergic ones [1, 8]. Moreover, there is a local pineal RAS that modulates the synthesis of melatonin, which represents the main hormonal output of the pineal gland [9, 10]. The RAS is classically involved in cardiovascular and metabolic pathophysiology while melatonin deals with circadian rhythms. In this paper, we aimed at evidencing interference at several levels between the RAS and melatonin to modulate cardiovascular and metabolic pathophysiology (Table 1).

## 2. Angiotensin-Melatonin Axis

**Figure 1:**
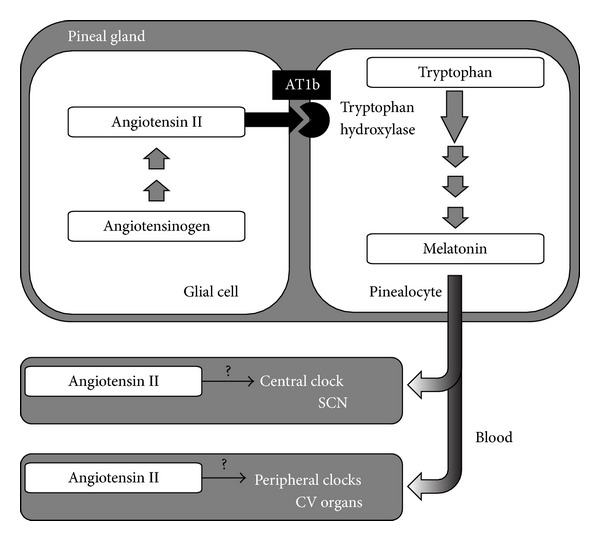
Pineal renin-angiotensin system (RAS) interacts with melatonin synthesis. Angiotensin II, produced from angiotensinogen produced by glial cells, acts on AT1b receptors present on pinealocytes to stimulate tryptophan hydroxylase, which is the rate-limiting enzyme in melatonin synthesis. Both angiotensin and melatonin may interact to regulate rhythmicity either centrally in the suprachiasmatic nucleus (SCN) or peripherally in clocks present in several cardiovascular organs.

The postulation of a local RAS in the brain has led to discovery of brain-specific roles of angiotensin II (Ang II). A local production of active angiotensins has been documented in several brain nuclei and regions. Of these, the two neuroendocrine glands situated in the brain possess high levels of Ang II-forming activities [9]. This led us to postulate the presence of a local RAS in the pineal gland. Angiotensinogen, the precursor of the RAS, has been identified in pineal glial cells while the receptors type AT1b are localized in pinealocytes [10]. As part of the enzymatic cascade producing Ang II, we identified angiotensin converting enzyme (ACE) and chymase but not renin, indicating the existence of nonrenin pathways [9, 10]. By utilizing both pharmacological and transgenic strategies, we could demonstrate that locally produced Ang II in the pineal can modulate the melatonin synthesis [11]. Melatonin represents the main hormonal output of the pineal gland and it is considered as an important modulator of circadian rhythms. Our studies indicate that Ang II acts on the pinealocyte AT1 receptors to influence the synthesis and activity of tryptophan hydroxylase (TPH), the rate-limiting enzyme of melatonin synthesis. The demonstration of a functional pineal RAS interfering with melatonin synthesis indicates that this may affect melatonin roles, such as in modulation of circadian rhythms (Figure 1).

The circadian system comprised of a group of specialized genes is a key integrator of metabolism and behavior that synchronizes physiological processes. Circadian oscillators are present not only in the suprachiasmatic nucleus (SCN) which is considered to be the master clock but also in peripheral tissues, including cardiovascular organs [12]. Several animal studies are identifying roles for clock genes in cardiovascular and metabolic physiology and pathophysiology [13]. Genetic manipulation of clock genes in transgenic mouse models has uncovered new functions of internal clocks in pathogenesis of cardiovascular diseases [14]. Since our and other studies point to an important role of the RAS in the modulation of circadian rhythms of blood pressure [15, 16], an interaction with the genes governing circadian clock of cardiovascular tissues might be conceivable. An interaction between the RAS and the circadian system has been suggested to contribute to the development of inverted BP profile in transgenic rats harboring the mouse Ren-2 renin gene, TGR(mREN)27 [17, 18].

The interaction between angiotensin and melatonin both centrally and peripherally in hypertension and cardiovascular diseases has to be studied. At pineal level, the challenge is to understand in a hypertensive patient with elevated levels of angiotensin how melatonin release is affected: is there a feedback loop of high levels of circulating angiotensin impacting on either potentiating more melatonin release to counteract hypertension, or if there is a lack of effect, that is, resistance to central angiotensin, that resets the pineal gland axis and reduces melatonin output, which then may further potentiate hypertension. Further studies are necessary to decipher and dissect possible interactions between the RAS, melatonin, and the clock genes governing circadian clock.

## 3. Antagonistic Effects of Angiotensin and Melatonin in Cardiovascular and Metabolic Systems

### 3.1. Cardiovascular System

A cardiovascular role of melatonin has been suggested already 40 years ago by the description of a pinealectomy-induced experimental hypertension model [19–21]. Melatonin receptors are present in the vasculature and mediate vascular constriction and vasodilation through MT1 and MT2 receptors, respectively [22]. Melatonin administration generally induces a decrease in blood pressure [22]. One possible mechanism contributing to the melatonin hypotensive effects is through its sympatholytic properties [23]. On the other hand, a marked reduction of circulating melatonin has been observed in cardiovascular diseases [24]. These findings suggest antagonistic activities of angiotensin and melatonin in the cardiovascular system (Table 1). The mechanisms by which melatonin is antagonizing Ang II actions in cardiovascular and metabolic diseases are comprising its antihypertensive, antioxidant, and anti-inflammatory functions [24]. Melatonin has direct free radical scavenging and indirect antioxidant activity. Through these marked antioxidant properties, melatonin has cardioprotective effects, in particular in myocardial damage after ischemia-reperfusion [25].

### 3.2. Circadian Rhythms of the Cardiovascular System

Multiple clinical studies have implicated blood pressure (BP) and heart rate (HR) variability in the diagnosis and prognosis of arterial hypertension and cardiovascular diseases. In healthy individuals, there is a circadian variation of BP with a nocturnal fall of 10%–20% during the sleep period [26]. In hypertensive patients, this circadian rhythm may disappear or even become inverted. Therefore, according to the BP circadian alterations, patients have been classified as “dippers” when the mean nighttime BP is ≥10% lower than the mean daytime BP, as “nondippers” when the reduction is <10% or as “risers” when it is higher [27]. Nondippers and risers are at an increased risk for target organ damage and cardiovascular events [28, 29]. Moreover, a circadian pattern becomes quite obvious in the occurrence of acute cardiovascular diseases, such as ischemia, infarction, stroke, and sudden death, and new chronotherapeutic approaches in antihypertensive therapy are trying to exploit the knowledge of circadian rhythms in order to reduce these events [29]. Therefore, a better understanding of the molecular biology and pathophysiology of nondipper hypertension will lead to a better understanding of the disease and possibly lead to new diagnostic tools or therapeutic strategies. Further studies investigating the molecular mechanisms of the circadian regulation of the cardiovascular system should hopefully reveal new diagnostic tools or treatment algorithms for disease.

**Figure 2:**
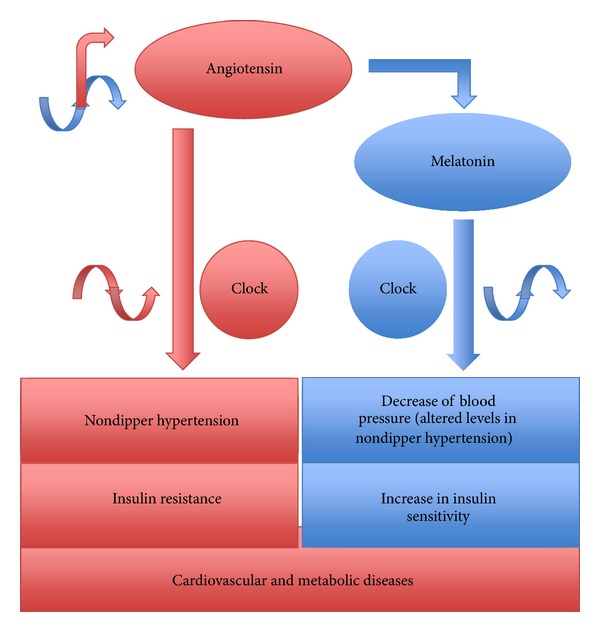
Angiotensin versus melatonin in cardiovascular and metabolic diseases. An increase in angiotensin induces nondipper/riser hypertension, which is characterized by a decrease in melatonin. Angiotensin and melatonin have opposing effects on insulin sensitivity.

Our group was the first to do demonstrate that chronic Ang II infusion may induce a shift in the circadian BP rhythm (Figure 2) [16]. Ang II infused subcutaneously at doses of up to 250 ng/kg per minute that does not produce direct vasoconstriction is described as “slow pressor” or “subpressor” and can induce a gradual increase of BP. Chronic infusion (days to weeks) of subpressor Ang II subcutaneously induces nondipper hypertension similarly with the renovascular and other forms of human hypertension where the circadian variation of blood pressure is altered [16, 30, 31]. Alterations in the circadian BP rhythm are not synchronized with alterations of heart rate or locomotor activity, contributing to the concept that the circadian variability in blood pressure and heart rate are differentially regulated [15, 16]. We further hypothesized that the brain RAS might be involved in the Ang II-induced BP circadian shift. To test this hypothesis, we studied a transgenic rat that has reduced angiotensinogen levels in the brain through expression of an antisense RNA against angiotensinogen, induced by means of the astrocyte-specific glial fibrillary acidic protein promoter [32]. Ang II infusion in the TGR(ASrAOGEN) transgenic rats did not induce a BP circadian shift, indicating that peripheral RAS interacts with the brain RAS to induce not only hypertension [2] but also a BP circadian shift [15, 16]. We employed the TGR(ASrAOGEN) to investigate if the brain RAS is involved in circadian rhythm reentrainment to light phase shifts. The BP and HR acrophases (peak time of curve fitting) in TGR(ASrAOGEN) rats readjusted to light shifts significantly slower than in control (Sprague-Dawley) rats [15, 16]. However, the acrophases of locomotor activity changed similarly in both strains. These data suggest that treatment with RAS blockers with high penetrability of the blood-brain barrier (such as candesartan and valsartan) could slow the resynchronization of cardiovascular system in jet-lag conditions of travelers adapting to a new time zone.

Research on the circadian actions of Ang II has preoccupied several research groups. Among these, the group of Lemmer has provided several lines of evidence on the importance and the mechanisms regulating cardiovascular circadian rhythms [29]. Lemmer et al. provided significant insights in the pathophysiology of a transgenic rat model, TGR(mREN2)27 [17, 18]. The TGR(mREN2)27 is a well-characterized model of malignant hypertension due to an overactive RAS as it harbors the mouse salivary gland renin gene (mREN2). The TGR(mREN2)27 not only become hypertensive and develop target-organ damage but also exhibit an inverted circadian rhythm of BP, which makes them a valuable model to further study the molecular biology of circadian rhythms. However, detailed mechanisms responsible for the Ang II-induced nondipper hypertension are still not well understood. The circadian rhythms that are a characteristic of most of the physiological parameters are governed by biological clocks. Ablation of the SCN of the hypothalamus that serves as the main zeitgeber for such circadian rhythms eliminates BP, HR, and locomotor activity [33, 34]. Ang II might interact directly or through melatonin to influence the 24-h rhythmic expression of clock genes in SCN (Figure 1). Evidence on a mutual relationship between melatonin and circadian oscillators has recently been reviewed by Hardeland [35]. Also, an impaired nocturnal melatonin secretion has been detected in nondipper hypertensive patients (Figure 2) [36]. Besides melatonin, the brain RAS might influence the central circadian clock present in the hypothalamic SCN either directly or through vasopressin that is another hormone with demonstrated roles in circadian rhythms [8, 11, 37]. 

Ang II and melatonin might interact with circadian oscillators present not only in the SCN but also in peripheral tissues, including cardiovascular organs (Figure 1) [12]. Several animal studies are identifying roles for clock genes in cardiovascular physiology [13]. Genetic manipulation of clock genes in transgenic mouse models has uncovered new functions of internal clocks in pathogenesis of cardiovascular diseases [14]. Recent evidence is suggesting that a disruption of central or peripheral clocks may contribute to the progression of cardiovascular diseases [38]. Researching for further insights on the roles of biological clocks in cardiovascular organs shall provide acumens into the relevance of the circadian rhythms in cardiovascular pathology.

### 3.3. Metabolic Syndrome, Insulin Resistance, and Diabetes

Several lines of evidence including successful therapies with drugs acting on RAS are demonstrating roles of RAS in diabetes mellitus and metabolic syndrome. Putnam et al. recently reviewed the accumulating evidence describing the RAS as a “target of and contributor to dyslipidemias, altered glucose homeostasis, and hypertension of the metabolic syndrome” [39]. Angiotensin II causes insulin resistance through activation of the AT1R and increased production of mineralocorticoids [40]. However, the underlying mechanisms of Ang II leading to insulin resistance remain to be fully elucidated. Melatonin on the other hand induces an increased insulin sensitivity [35]. Evidence for a link between melatonin and insulin came from pinealectomized animals that develop diabetogenic syndrome characterized by insulin resistance and a 50% reduction of GLUT4 in adipose and muscular tissue [41]. Moreover, it was demonstrated that the absence of melatonin in pinealectomized animals impairs the temporal organization of several metabolic functions associated to the carbohydrate metabolism, such as daily insulin secretion, adaptation to starvation, and exercise [42–44]. This dramatic picture can be partially or totally restored by melatonin reposition or restricted feeding [45, 46]. Melatonin acting through MT_1_ membrane receptors is able to induce insulin receptor phosphorylation at the same time that mobilizes several intracellular transduction steps in common to insulin signaling [47]. Melatonin is able to restore insulin sensitivity and regulates food ingestion and body weight and abdominal adiposity in old rats [48].

Not only melatonin regulates insulin but also insulin can act on *in vitro* pineal glands potentiating the noradrenergic-induced melatonin synthesis, regulating the activity of the enzymes tryptophan hydroxylase and N-acetyltransferase through after-transcriptional mechanisms [49]. 

The first reports on diabetes and melatonin production showed that diabetic rats and mice, chemically-induced by alloxan (ALX) or streptozotocin (STZ), presented a decrease in melatonin synthesis and plasma levels [50, 51], although Champney et al. [52] reported no alterations in the level of melatonin in diabetic rats. Melatonin was also observed to suppress the onset of type 1 diabetes in nonobese mice, while pinealectomy had the opposite effect [53]. In type 2 diabetic patients and diabetic Goto Kakizaki rats a decreased serum melatonin level was observed [54]. In STZ-induced diabetic animals, melatonin was shown to decrease serum lipid oxidation [55] and protein glycosylation [56], as well as regulate the activity of antioxidant enzymes, improving the protection against the oxidative damage caused by diabetes [57–59]. Despite that, melatonin was not able to normalize hyperglycemia and/or body weight in these animals [55, 60, 61] and lower levels of the indolamine were observed in peripheral tissues like pancreas, kidney, spleen, and duodenum [62].

## 4. Conclusions and Perspectives

Accumulating evidence suggests that not only angiotensin interferes with melatonin synthesis and release but also both hormones interact at several levels having opposing effects in cardiovascular and metabolic pathophysiology (Table 1). Furthermore, evidence is indicating that not only melatonin but also angiotensin may interfere with circadian rhythms. The intimal regulatory mechanisms of interference between the two systems both centrally and peripherally, at synthesis and action levels, in homeostatic and disease conditions await further investigation. On how peripheral RAS interacts with the pineal RAS in hypertension and cardiovascular disease and if the sympathetic nervous system or other systems are involved in this interaction are still open questions.

Contemporary progress in chronobiology directs to an important role of clock-associated genes in the progression of cardiovascular and metabolic diseases. Since angiotensin appears to be involved in the modulation of circadian rhythms of blood pressure, an interaction with the clock genes seems likely. Therefore, we believe that further research on the molecular biology of circadian alterations involving interactions between angiotensin, melatonin, and clock genes may have an impact on cardiovascular and metabolic pathophysiology leading to new chronotherapeutic strategies.

## Figures and Tables

**Table 1:** Opposing roles of angiotensin II and melatonin in cardiovascular and metabolic pathophysiology.

Angiotensin	Melatonin
Blood pressure: direct effects	
Increase, vasoconstriction	Decrease, vasodilation

Blood pressure: circadian rhythm	
Nondipper/riser hypertension	Decreased levels in nondipper hypertension, chronobiotic

Central clock: suprachiasmatic nucleus	
Precursor and receptors present	Receptors present

Sympathetic nervous system	
Stimulation	Sympatholytic

Oxidative stress	
Increase	Decrease

Inflammation	
Increase	Decrease

Central clock: suprachiasmatic nucleus	
Precursor and receptors present	Receptors present

Insulin	
Insulin resistance	Increase in insulin sensitivity
